# Differential miR-346 and miR-582-3p Expression in Association with Selected Maternal and Fetal Complications

**DOI:** 10.3390/ijms18071570

**Published:** 2017-07-19

**Authors:** Pei-Yin Tsai, Sheng-Hsiang Li, Wan-Ni Chen, Hui-Ling Tsai, Mei-Tsz Su

**Affiliations:** 1 Department of Obstetrics and Gynecology, National Cheng Kung University Hospital, College of Medicine, National Cheng Kung University, Tainan 704, Taiwan; tsaipy@mail.ncku.edu.tw (P.-Y.T.); cuteyling@gmail.com (H.-L.T); 2 Institute of Clinical Medicine, College of Medicine, National Cheng Kung University, Tainan 704, Taiwan; shlin922@mail.ncku.edu.tw; 3 Department of Public Health, College of Medicine, National Cheng-Kung University, Tainan 704, Taiwan; 4 Biostatistics Consulting Center, National Cheng Kung University Hospital, Tainan 704, Taiwan; n119555@mail.hosp.ncku.edu.tw

**Keywords:** miR-346, miR-582-3p, preeclampsia, preterm delivery, intrauterine growth restriction

## Abstract

Several miRNAs are expressed in human gestational tissue, and some have been shown to be associated with placental dysfunction and complicated pregnancy outcomes. To investigate the roles of miR-346 and miR-582-3p in adverse obstetric events, we analyzed these 2 miRNAs in three samples (maternal blood, umbilical cord blood and placenta) obtained from pregnant women in four groups, including healthy control (*n* = 60), preeclampsia (*n* = 31), preterm delivery (*n* = 29) and small for gestational age (*n* = 19) patients. The expression levels of miR-346 and miR-582-3p in all included adverse obstetric outcome groups were significantly higher in the maternal plasma samples but lower in the placenta samples (all *p* value < 0.05). In addition, the miR-346 expression levels in fetal cord blood were also significantly lower in all of the included adverse obstetric outcome groups (all *p* < 0.05). Multivariate analysis of the three specimens after adjusting for maternal age and gestational age at delivery gave the same results. In conclusion, aberrant miR-346 and miR-582-3p expression level in pregnancy was associated with multiple maternal and fetal complications. Their differential expression in maternal blood, umbilical cord blood and placenta could be potential biomarkers or therapeutic targets for adverse obstetric outcomes

## 1 Introduction

MicroRNAs (miRNA) are small non-coding RNAs that regulate gene expression by binding and targeting specific mRNA transcripts for degradation or translational repression. They are well conserved and are thought to be a key evolutionarily component of genetic regulation [1]. Most miRNAs are multifunctional and play critical roles in a variety of cellular and physiological activities, such as cell growth, differentiation, apoptosis, migration and invasion [2,3]. Their expressional dysregulation has been shown to be important in many diseases, including cancer development and metabolic diseases [4,5].

The role of miRNAs in human pregnancy remains poorly understood. Several studies have identified miRNA profiles associated with pregnancy that are present in the placenta and in maternal circulation throughout gestation [6,7]. The biological roles of miRNAs can be very different from one tissue to another. Some specific placental miRNAs have been shown to be associated with embryo implantation, recurrent abortion and placental development [8,9,10], and some miRNAs in maternal circulation are associated with adverse pregnancy outcomes, such as ectopic pregnancy, molar pregnancy, gestational diabetics, intrauterine fetal growth restriction (IUGR) and preeclampsia [11,12,13,14]. Our previous in vitro result showed that miR-346 and miR-582-3p regulated trophoblast cell motility through inhibiting endocrine gland-derived vascular endothelial growth factor (EG-VEGF) [15]. EG-VEGF is an important molecular signal for embryo implantation and placenta development [16,17], and several studies also showed its association with multiple gestational complications [17,18]. However, the clinical roles of miR-346 and miR-582-3p in human pregnancy have never been explored.

Although evidence is emerging that abnormal expression of miRNAs is associated with pregnancy complications, most studies investigate a single type of specimen from pregnant women. In this study, we compared the individual expression levels of miR-346 and miR-582-3p in three specimen types, including maternal peripheral blood, fetal cord blood and placenta from pregnant women and further evaluated their association with multiple adverse obstetric outcomes, including preeclampsia, preterm delivery, and small for gestational age (SGA). The differential expression patterns of the miRNAs in the three gestational specimens show potential roles in adverse obstetrical outcomes and may be useful for future clinical diagnoses and therapeutic approaches. 

## 2. Results

### 2.1. Clinical Characteristics of Included Groups and Sample Collection

In total, we collected a trio of samples-maternal peripheral blood, fetal cord blood and placenta-from 126 pregnant women at the National Cheng-Kung University Hospital, Taiwan. Among these pregnant women, there were 60 healthy controls and 31, 29, and 19 cases of preeclampsia, preterm delivery, and SGA, respectively. There were no patient duplication in the groups of preeclampsia and preterm delivery. The complete collection rate of all 3 samples (maternal peripheral blood, fetal cord blood and placenta) from the subjects was more than 90–95% in each group. The C-section rates were 35% in the control group, 81% in the preeclampsia, 52% in the preterm delivery, and 47% in the SGA. The clinical characteristics of the included groups are summarized in Table 1. The most significant differential clinical characteristic between the case and control groups was the gestational age at delivery. Fetal birth weight (BW), placenta weight and newborn Apgar score are all related to the gestational age. After comparing gestational age-matched subjects (>37 weeks of gestation) in the adverse obstetric outcome groups (preeclampsia and SGA) with healthy controls, the other possible clinical confounding factors (fetal BW, placenta weight and newborn Apgar score) were all eliminated in group of preeclampsia (Table S1).

### 2.2. The Expression of miR-346 in Maternal Peripheral Blood, Fetal Cord Blood and Placenta 

**Figure 1:**
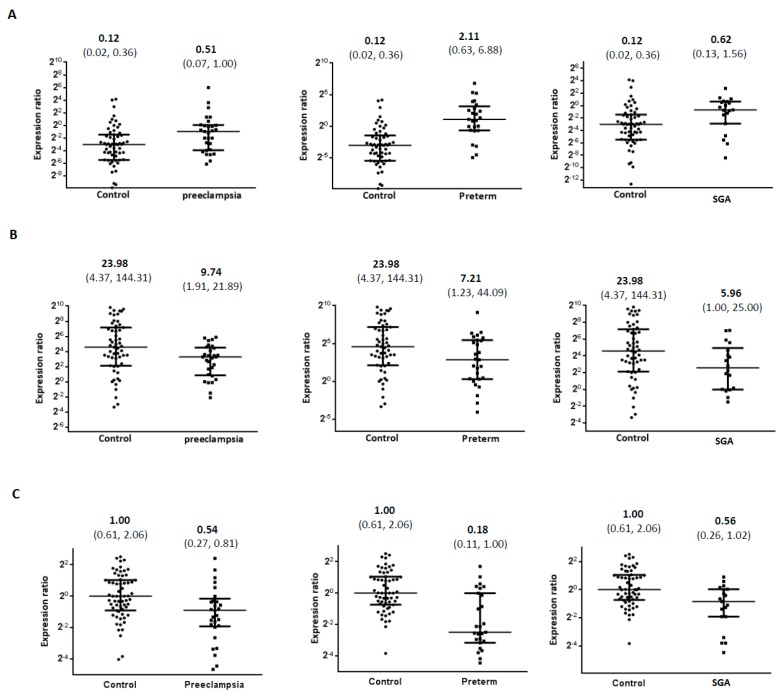
Comparison of miR-346 expression levels between compromised and healthy pregnancies in three gestational specimens. MiR-346 expression levels [median (interquartile range)] of maternal plasma (**A**) are significantly higher in every adverse obstetric outcome group (preeclampsia, preterm delivery, and small for gestational age) evaluated in this study (all *p* values < 0.05), whereas its expression levels in fetal cord plasma (**B**) and in the placenta (**C**) are lower in the adverse obstetric outcome groups.

To investigate the role of miR-346 in obstetric disorders, we analyzed the expression levels of miR-346 in three sample types (maternal plasma, fetal cord plasma and placenta) from women with preeclampsia, preterm delivery, and SGA, and compared their concentrations with healthy controls. U6 was used as an interval control in the present study, and the Ct values of U6 in the control and case groups were 34.01 ± 2.65 vs. 33.86 ± 2.77 in maternal plasma, 33.88 ± 2.96 vs. 32.80 ± 2.68 in fetal cord plasma, and 25.88 ± 1.90 vs. 25.46 ± 2.32 in placenta (mean ± SD, all *p* values > 0.05). All the miRNA expression levels in the tables and figures were presented as the values of 2^−ΔΔ*C*t^ from quantitative real-time PCR (qRT-PCR). The miR-346 expression levels in maternal plasma in the third trimester were variable, but its levels in all groups with obstetric complications (preeclampsia, preterm delivery and SGA) were higher than those of healthy controls (Table 2 and Figure 1A). Compared to the levels in healthy controls, we found a 4.2-fold increase in maternal plasma miR-346 in the preeclampsia group (median: preeclampsia, 0.51, vs. controls, 0.12, *p* < 0.05), a 17.6-fold increase in the preterm delivery group (median: preterm, 2.11, vs. controls, 0.12, *p* < 0.05), and a 5-fold increase in the SGA group (median: SGA, 0.62, vs. controls, 0.12).

Although miR-346 expression levels in fetal cord blood and placenta are also widely variable, their expression levels were both lower in these two specimen types for all groups with obstetric complications (Table 2 and Figure 1B,C). Compared to the levels in healthy controls, we found a 2.46-fold decrease in miR-346 in fetal cord plasma in the preeclampsia group (median: preeclampsia, 9.74, vs. controls, 23.98, *p* < 0.05), a 3.3-fold decrease in the preterm delivery group (median: preterm, 7.21, vs. controls, 23.98), and a 4.0-fold decrease in the SGA group (median: SGA, 5.96, vs. controls, 23.98). The miR-346 expression levels in placental tissue were less variable than those of maternal and fetal cord plasma. For miR-346 expression in placenta, we found a 1.9-fold decrease in miR-346 in the preeclampsia group (median: preeclampsia, 0.54, vs. controls, 1.00, *p* < 0.05), a 5.6-fold decrease in the preterm delivery group (median: preterm, 0.18, vs. controls, 1.00, *p* < 0.05), and a 1.8-fold decrease in the SGA group (median: SGA, 0.56, vs. controls, 1.00, *p* < 0.05). To eliminate the possible confounding factors of maternal age and gestational age at delivery, we analyzed the data using the multivariate linear regression model. After adjusting for maternal age and gestational age at delivery, the miR-346 expression in three specimens still significantly associated with multiple adverse pregnancy outcomes (Table 3).

### 2.3. The Expression of miR-582-3p in Maternal Peripheral Blood, Fetal Cord Blood and Placenta 

**Figure 2:**
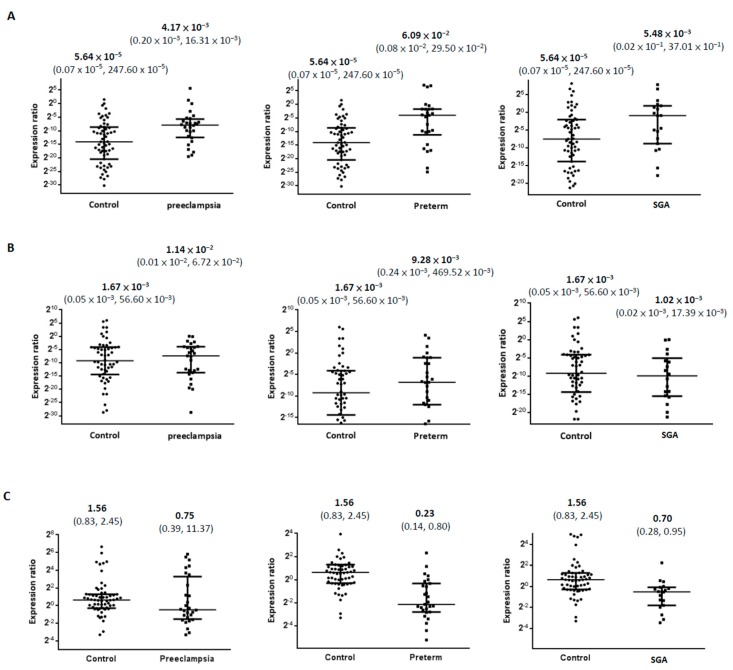
Comparison of miR-582-3p expression levels between compromised and healthy pregnancies in three gestational specimens. miR-582-3p expression levels [median (interquartile range)] of maternal plasma (**A**) are significantly higher in groups of preeclampsia and preterm delivery (*p* values < 0.05). Although miR-582-3p expression levels in fetal cord plasma (**B**) are not significantly different, its expression level in the placenta (**C**) is lower in the groups of preterm delivery and SGA than in the healthy controls (*p* values < 0.05).

We analyzed the expression levels of miR-582-3p in the trio of samples from women in the healthy control, preeclampsia, preterm delivery, and SGA groups to investigate its role in obstetric disorders. The miR-582-3p expression levels of maternal and fetal cord plasma were lower and much more variable than those of miR-346, and the differences in concentration ranged from 10- to 1000-fold in each group. Nonetheless, the miR-582-3p expression levels in maternal plasma were significantly higher in preeclampsia and preterm delivery groups than in the controls (*p* < 0.05, Table 2 and Figure 2A). Compared with the levels in the controls, the miR-582-3p expression levels in maternal plasma were increased 73.9-fold in the preeclampsia group (median: preeclampsia, 4.17 × 10^−3^, vs. controls, 5.64 × 10^−5^, *p* < 0.05), and 1079-fold in the preterm delivery group (median: preterm, 6.09 × 10^−2^, vs. controls, 5.64 × 10^−5^, *p* < 0.05).

The miR-582-3p expression levels in placenta were significantly lower in preterm delivery and SGA groups than in the controls (*p* < 0.05, Table 2 and Figure 2C). Compared with the levels in the controls, the miR-582-3p expression levels in placenta were decreased 6.78-fold in the preterm delivery group (median: preterm, 0.23, vs. controls, 1.56, *p* < 0.05), and 2.23-fold in the SGA group (median: SGA, 0.70, vs. controls, 1.56, *p* < 0.05). After adjusting for gestational age at delivery and maternal age, the expression analysis results of miR-582-3p in maternal plasma and placenta remained significantly different between the healthy and compromised pregnancy outcomes (Table 3).

## 3. Discussion

In this study, we investigated the expression of miR-346 and miR-582-3p in trios of samples, maternal blood, fetal cord blood and placenta, in order to evaluate the relationship of these specific miRNAs among the three sample types and to further evaluate the association between miRNAs and adverse obstetric events. We found that the miRNA expression patterns of pregnant women were different among these three sample types. Moreover, miR-346 and miR-582-3p were shown to be associated with every adverse obstetric outcome explored in this study (preeclampsia, preterm delivery and SGA). Notably, miR-346 and miR-582-3p expression levels in the maternal plasma of patients with adverse obstetric events were all upregulated compared with healthy controls, whereas their expression levels were all downregulated in the placenta and/or fetal cord blood of patients with adverse obstetric outcomes.

Limited work to date is available to provide information about the effect of one specific miRNA on different cells or tissues as well as the association between miRNAs from different specimens and human disorders. However, the role of one specific miRNA in different cells or tissues is generally believed to be different as one mRNA may be targeted by multiple different miRNAs, and a single miRNA may regulate several mRNAs [19]. When evaluating the role of miRNAs in adverse pregnancy outcomes, most studies investigated a single specimen type (placenta or maternal blood), and only some studied both. Some miRNAs from placenta and maternal plasma showed similar aberrant expression profiles in complicated pregnancies [20,21], while some did not [22]. The differential expression patterns of miRNAs in maternal circulation and from placenta were also reported in another study. Mouillet et al. reported that the miRNA levels they analyzed (12 miRNAs) increased 1.84-fold in the plasma of women with a fetal growth restriction (FGR) complication but decreased 24% in FGR placenta samples compared with the values in uncomplicated pregnancies [22]. The cell types analyzed in placenta include syncytiotrophoblasts/cytotrophoblasts, mesenchymal cells, Hofbauer cells, fibroblasts, and fetal vascular cells [23]. While the cell origin of miRNA detected in maternal or fetal plasma is much more complex. Circulating miRNAs could be passively released by broken cells (derived from cell inflammation, necrosis or apoptosis), or actively cell secreted microvesicles (exosomes or shedding vesicles), or directly in complex with binding proteins (RNA-binding proteins, lipoproteins and Argonaute proteins) [24]. Therefore, their cell origins could be any cells secreted or derived from maternal or fetal circulation systems and are distant from their target sites. The miRNA expression profile is cell- and tissue-specific, and the composition appears to differ greatly from their respective donor cells after changing to another environment [25]. Although various cell origins of circulating miRNA in maternal and fetal cord plasma, most studies focused on placental trophoblast and maternal/fetal endothelial cells [26,27,28].

The study of miRNAs in fetal cord blood and their effect on pregnancy or neonatal outcomes has not been explored thus far. In the placenta of fetal-maternal interface, fetal syncytiotrophoblast in the intervillous space is direct contact with maternal blood from spiral arteries. The effect of miRNA from fetal cord blood on maternal pregnancy health could exert through this interface. Very small amount of fetal cells, cell-free fetal component and placenta-derived exosomes could be detected in maternal circulation since early gestation [26,29,30], and these fetal- or placenta-derived substances can transfer microRNAs or other molecules to distant target of maternal system and influence maternal health. In turn, maternal gestational health also has direct impact on intrauterine fetal biological function. Several studies have shown that trophoblast debris, cell-free fetal DNA, placenta-derived exosomes and fetal endothelial cells may predispose several adverse maternal and fetal outcomes, such as preeclampsia, gestational diabetes and fetal growth restriction [26,27,28,30]. Considering the increasing evidence that miRNAs from maternal plasma and placenta are involved in various fundamental biological processes, fetal cord miRNAs should have a significant impact on maternal gestation and regulation of fetal/neonatal growth and development. Besides, fetal cord blood contents can be fluctuated by maternal or child biological condition (oxygen, pH value, nutrients) depending on the source of cord blood [31], and therefore miRNA expression in fetal cord artery may be different from the one in fetal cord vein. In this study, we analyzed the miRNA expression from fetal cord blood from its vein route, and the miR-346 levels in cord plasma were all significantly downregulated in the patient groups with preeclampsia, preterm labor, and SGA, suggesting its possible roles in these complicated pregnancies; however, the functional impact of fetal cord miRNAs in each gestational disorder and their underlying mechanism require further exploration.

More than 500 miRNAs are expressed in human placenta, and their roles are suspected to regulate placental development and trophoblast cell functions, such as trophoblast proliferation, differentiation, apoptosis, invasion, and angiogenesis [32]. Several miRNAs were reported to be associated with defective placentation and complicated pregnancies [33,34], and aberrant expression of some miRNAs in the placenta or maternal plasma, such as miR-210 and miR-518b, were associated with both preeclampsia and SGA [20,21,35]. In this study, miR-346 and miR-582-3p in maternal plasma or/and in the placenta were associated with preeclampsia and SGA. Considering that some miRNA levels may change as pregnancy progresses, we adjusted this factor and the results gave the same conclusion, which suggests that miR-346 and miR-582-3p are not affected by gestational age in the late third trimester. The similar expression trends of miR-346 and miR-582-3p in maternal plasma and in the placenta, and their association with these two disorders (preeclampsia and SGA), suggests that these two miRNAs may be involved in the disease pathophysiologies and further supports that these two pregnancy-related disorders may share similar mechanisms.

Apart from our previous publication, the roles of miR-346 and miR-582-3p in human pregnancy have never been explored [15]. Our results showed that miR-346 and miR-582-3p inhibit trophoblast cell invasion and migration through targeting the 3’UTR of EG-VEGF to downregulate gene expression [15]. EG-VEGF was shown its association with multiple gestational disorders, such as recurrent abortion, preeclampsia, IUGR, gestational trophoblast disease, and placental accreta in several studies [17,18]. Whether the roles of miR-346 and miR-582-3p in disease formation during human pregnancy are through regulating EG-VEGF or interacting with a group of associated genes, further experimental and clinical studies are required to validate the effects of miRNAs in EG-VEGF-associated obstetric disorders.

The strength of this study is that we collected three specimens from pregnant women at delivery and provided evidence that one miRNA could be expressed differentially from one to another gestational specimen. Different expression patterns of one specific miRNA in multiple specimens reported in different studies would be attributed to the diversity of study components (different ethnicity, study subjects, gestation age, analysis method, etc.), but this is not a concern in one study of the same population. Surprisingly, the two miRNAs in the present study had the same expression trends (upregulation or downregulation) in the different specimen types among the various groups of patients with compromised gestational disorders. Nevertheless, there are some limitations in this study. First, the exact effect of the two miRNAs in each individual adverse obstetric outcome is lacking. The second limitation is the relatively small size of each compromised pregnancy group. The third is the choice of internal control, U6, which could be a suboptimal reference gene. Although no significant Ct-value difference between control and case groups in our data, some studies showed its instability during freeze-and-thaw cycles [36,37]. There is so far lack of a consensus suitable reference gene for all miRNA quantification. Before a universal reference gene was identified, the better solution would be using more than one internal control to produce more accurate and reliable normalization of q-PCR data in each cell type and experimental condition. Even though, we have provided preliminary but promising data in the present study, and future studies could build on our results to explore the underlying pathophysiologies or to apply these miRNAs in adverse pregnancy outcomes in clinical practice.

## 4. Materials and Methods

### 4.1. Study Population and Sample Collection

This study was approved by the Institutional Review Board of the National Cheng Kung University Hospital (Tainan, Taiwan) (#A-ER-104-209), and informed consent was obtained from study participants between December 2015 to December 2016. A case-control study was conducted to examine the miRNA expression levels in maternal peripheral blood, fetal cord blood and placenta from patients in the following groups: (1) healthy controls (*n* = 60); (2) preeclampsia (*n* = 31); (3) preterm delivery (*n* = 29); and (4) small for gestational age (SGA) (*n* = 19). Preeclampsia was defined as hypertension (systolic blood pressure ≥140 mm Hg or diastolic blood pressure ≥90 mmHg on at least 2 occasions taken ≥4 h apart) and proteinuria (≥300 mg in a 24-hour urine collection or 1 dipstick measurement of ≥+2) occurring after 20 weeks of gestation in a previously normotensive woman. Preterm delivery was defined as regular uterine contraction with cervical changes that resulted in delivery before 37 weeks of gestation. SGA was defined as a birth weight below the 10th percentile for the fetus’ gestational age with known or unknown causes (idiopathic, preeclampsia, gestational diabetes, etc.).

Maternal blood samples were collected prepartum after hospital admittance for delivery (within hours to 2 days before delivery) and were placed in tubes containing ethylenediaminetetraacetic acid (EDTA). Placental tissue and cord blood samples were obtained immediately following delivery. Placental tissue samples were retrieved from the middle layer of cross-section without contacting maternal and fetal sides, cut into small pieces with scissor, placed in microcentrifuge tubes (SSI, Lodi, CA, USA), and stored in −80 °C before further analysis. Fetal cord blood samples were retrieved from its vein route and immediately placed in tubes containing EDTA.

### 4.2. miRNA Isolation and Quantitative Real-Time PCR (qRT-PCR)

To harvest cell-free plasma from the maternal and fetal cord blood samples, we centrifuged the whole blood samples twice at 1200× *g* for 10 min at room temperature. Plasma and placenta samples were stored at −80 °C until further processing. Prior to RNA purification, 25 mg of placental tissues were homogenized in 600 μL of Tri reagent^®^ with bead beating (ZR BashingBead Lysis Tubes, ZYMO, Irvine, CA, USA) in a high-speed homogenizer for 15–20 min. Total RNA was extracted from 200 μL of plasma samples and 25 mg of placental tissue using a commercial column-based system following the manufacturer’s instructions (Direct-zol RNA Miniprep kit, ZYMO, Irvine, CA, USA). To minimize DNA contamination, we treated the samples with 5 μL of DNase I in the columns for 15 min at 25–30 °C.

Each miRNA was reverse transcribed using the TaqMan MicroRNA Assay containing microRNA-specific stem-loop RT primers and a TaqMan MicroRNA Reverse Transcription Kit (Applied Biosystems, Foster City, CA, USA) in a total reaction volume of 15 μL according to the manufacturer’s instructions. Reverse transcriptase reactions were performed using a GeneAmp PCR system 2700 (Applied Biosystems, Foster City, CA, USA). MiRNAs were quantified using the TaqMan Universal PCR Master Mix (Applied Biosystems, Foster City, CA, USA) and individual miRNA primers and hydrolysis probes (Applied Biosystems, Foster City, CA, USA) using the StepOne Plus instrument (Applied Biosystems, Foster City, CA, USA). The threshold cycle (*C*_t_) was determined using the instrument default threshold settings. U6 was used as an endogenous control, and the relative expression levels of each miRNA were calculated using the 2^−ΔΔ*C*t^ method.

### 4.3. Statistical Analyses

Continuous variables of patient characteristics were defined as means (±SD, standard deviation), while categorical variables were presented as percentages. According to the distribution pattern of continuous variables, comparisons between groups were performed by one-way ANOVA (or Kruskal-Wallis test, available online: http://www.statisticssolutions.com/kruskal-wallis-test/) with post hoc test. Comparisons between categorical data of patient characteristics were using the chi-square test or Fisher’s exact test. The miRNA expression levels of healthy controls were compared separately with that of patients in each group. All experimental assay values are expressed as the median ± interquartile range (IQR), and miRNA differences between the two groups were calculated with the Kruskal-Wallis with post hoc test. The relationship between miRNA level and adverse pregnancy outcome was examined using multivariate linear regression adjusting for maternal age and gestational age at delivery. Levels of miRNA were natural log-transformed to normality. Regression coefficients β along with their significance from the multiple linear regression analysis were also reported. Statistical analyses were performed using GraphPad Prism 5.0 (GraphPad, San Diego, CA, USA) and SAS 9.4 (SAS Inc., Cary, NC, USA). A two-tailed *p* value of <0.05 was regarded as statistically significant.

## 5. Conclusions

miRNAs are stable and differentially expressed in the gestational specimens investigated in this study. Aberrant miR-346 and miR-582-3p expression profiles in maternal plasma, fetal cord plasma and placenta samples were shown to be associated with preeclampsia, preterm delivery, and SGA. Before miR-346 and miR-582-3p being a non-invasive biomarker for adverse obstetric outcomes and a potential therapeutic target for preventing or treating compromised pregnancy, future studies with larger sample size in different population were needed to replicate our findings.

## Figures and Tables

**Table 1:** Characteristics of included study population.

Clinical Information	Normal Control (*n* = 60)	Preeclampsia (*n* = 31)	Preterm Delivery (*n* = 29)	Small for Gestational Age (*n* = 19)
Maternal Age (y)	31.33 ± 4.31	33.83 ± 5.77	32.04 ± 5.14	31.68 ± 7.21
Nulliparity (%)	56.7%	48.4%	34.5% *	73.7%
Gestational Age at Delivery (week)	38.87 ± 0.99	36.84 ± 2.30 *	34.73 ± 2.00 *	36.87 ± 3.01 *
Ethnicity (Chinese Hans %)	100%	100%	96.3%	89.5%
Cesarean Section	35.0%	80.7% *	51.9%	47.4%
BMI at Delivery (kg/m^2^)	26.47 ± 3.55	29.10 ± 5.60	26.04 ± 4.46	24.67 ± 3.16
Neonatal Outcome				
Birth Weight (g)	3192 ± 299.59	2622 ± 676.74 *	2214 ± 502.45 *	2173 ± 598.61 *
Sex (female %)	50%	50%	51.7%	57.9%
1 min Apgar Score	8.635 ± 0.71	8.063 ± 1.16 *	7.36 ± 1.04 *	7.938 ± 1.18
5 min Apgar Score	9.827 ± 0.43	9.469 ± 0.88	9.160 ± 0.75 *	9.438 ± 0.89
Placenta Weight (g)	631.4 ± 113.95	560 ± 158.32 *	562.1 ± 182.86 *	437.4 ± 113.32 *

Data are presented as means ± SD. Statistical test: Continuous variables used ANOVA with post hoc procedure; Categorical variables used chi-square test or Fisher’s exact test. * *p* value < 0.05.

**Table 2:** miR-346 and miR-582-3p expression values in three specimens of included study subjects.

Specimen Origin	Normal Control (*n* = 60)	Preeclampsia (*n* = 31)	Preterm Deliver (*n* = 29)	Small for Gestational Age (*n* = 19)
**miR-346**				
Maternal Plasma	0.12 (0.02, 0.36)	0.51 * (0.07, 1.00)	2.11 * (0.63, 6.88)	0.62 * (0.13, 1.56)
Fetal Cord Plasma	23.98 (4.37, 144.31)	9.74 * (1.91, 21.89)	7.21 (1.23, 44.09)	5.96 (1.00, 25.00)
Placenta	1.00 (0.61, 2.06)	0.54 * (0.27, 0.81)	0.18 * (0.11, 1.00)	0.56 * (0.26, 1.02)
**miR-582-3p**				
Maternal Plasma	5.64 × 10^−5^ (0.07 × 10^−5^, 24.76 × 10^−5^)	4.17 × 10^−3^ * (0.20 × 10^−3^, 16.31 × 10^−3^)	6.09 × 10^−2^ * (0.08 × 10^−2^, 29.50 × 10^−2^)	5.48 × 10^−3^ (0.02 × 10^−1^, 37.01 × 10^−1^)
Fetal Cord Plasma	1.67 × 10^−3^ (0.05 × 10^−3^, 56.60 × 10^−3^)	1.14 × 10^−2^ (0.01 × 10^−2^, 6.72 × 10^−2^)	9.28 × 10^−3^ (0.24 × 10^−3^, 469.52 × 10^−3^)	1.02 × 10^−3^ (0.02 × 10^−3^, 17.39 × 10^−3^)
Placenta	1.56 (0.83, 2.45)	0.75 (0.39, 11.37)	0.23 * (0.14, 0.80)	0.70 * (0.28, 0.95)

Data are presented as relative expression values of quantitative real-time PCR (2^−ΔΔ*C*t^) with median (IQR). Statistical test: Kruskal-Wallis test with post hoc procedure, and * denotes *p* value less than 0.05.

**Table 3:** Slope of log miR-346 and log miR-582-3p expression in three specimens of included study subjects.

Specimen Origin	Normal Control (*n* = 60)	Preeclampsia (*n* = 31)		Preterm Delivery (*n* = 29)		Small for Gestational Age (*n* = 19)	
		slope	*p* value	slope	*p* value	slope	*p* value
**miR-346**							
Log Maternal plasma	1	1.13 *	0.049	2.50 *	<0.001	1.16	0.068
Log Fetal cord plasma	1	−1.45 *	0.008	−1.81 *	0.006	−1.50 *	0.015
Log Placenta	1	−0.61	0.059	−1.53 *	<0.001	−0.99 *	0.007
**miR-582-3p**							
Log Maternal plasma	1	3.14 *	0.020	4.20 *	0.010	2.44	0.099
Log Fetal cord plasma	1	1.37	0.455	2.18	0.185	−0.36	0.812
Log Placenta	1	0.21	0.603	−1.67*	<0.001	−1.14 *	0.012

Statistical test: Linear regression adjusted for maternal age and gestational age at delivery. * denotes *p* value less than 0.05.

## Supplementary Materials

Supplementary materials can be found at www.mdpi.com/1422-0067/18/7/1570/s1.
