# Supplementary material for: Differential miR-346 and miR-582-3p Expression in Association with Selected Maternal and Fetal Complications
Source: Int J Mol Sci. 2017 Jul 19;18(7):1570. doi: 10.3390/ijms18071570 (PMC5536058; doi:10.3390/ijms18071570)
Supplement: Supplementary file 1 [file ijms-18-01570-s001.pdf]

Table S1. Characteristics of study participants with term pregnancy (gestational age> 37 weeks)

Clinical Information	Normal control (n = 60)	Preeclampsia (n = 15)	Small for gestational age (n = 11)
Maternal age (y)	31.33 ± 4.31	35.73 ± 5.64 *	32.73 ± 8.24
Gestational age at delivery (week)	38.87 ± 0.99	38.36 ± 1.12	38.77 ± 0.97
Ethnicity (Chinese %)	100%	100%	89.5%
BMI at delivery (Kg/m^2)	26.47 ± 3.55	29.12 ± 5.60	25.02 ± 3.42
Neonatal outcome			
Birth Weight (g)	3136 ± 505.11	2991.73 ± 571.86	2534.73 ± 244.78 *
1 min Apgar score	8.635 ± 0.71	8.6 ± 0.74	8.55 ± 0.73
5 min Apgar score	9.827 ± 0.43	9.86 ± 0.35	9.89 ± 0.33
Placenta weight	631.45 ± 113.95	603.87 ± 151.64	451.55 ± 56.19 *

Data are presented as means ± SD; statistical test: ANOVA with post hoc procedure, and * denotes p value less than 0.05
